# Zn-mobilizing bacteria improve shoot biomass and zinc content in wheat

**DOI:** 10.1093/femsec/fiag030

**Published:** 2026-04-13

**Authors:** Belay Berza, Fassil Assefa, Tesfaye Wubet, Undine Behrendt, Sizhong Yang, Paul Reim, Steffen Kolb

**Affiliations:** Department of Biology, College of Natural and Computational Sciences, DebreMarkos University, 269 Debre Markos, Ethiopia; RA Landscape Functioning, Leibniz Centre for Agricultural Landscape Research (ZALF), Eberswalder Str. 84, 15374 Müncheberg, Germany; Department of Microbial, Cellular and Molecular Biology, College of Natural and Computational Sciences, Addis Ababa University, 1176 Addis Ababa, Ethiopia; Helmholtz Centre for Environmental Research-UFZ, Department of community Ecology, 06120 Halle (Saale), Germany; German Centre for Integrative Biodiversity Research (iDiv) Halle-Jena-Leipzig, 04103 Leipzig, Germany; RA Landscape Functioning, Leibniz Centre for Agricultural Landscape Research (ZALF), Eberswalder Str. 84, 15374 Müncheberg, Germany; RA Landscape Functioning, Leibniz Centre for Agricultural Landscape Research (ZALF), Eberswalder Str. 84, 15374 Müncheberg, Germany; RA Landscape Functioning, Leibniz Centre for Agricultural Landscape Research (ZALF), Eberswalder Str. 84, 15374 Müncheberg, Germany; RA Landscape Functioning, Leibniz Centre for Agricultural Landscape Research (ZALF), Eberswalder Str. 84, 15374 Müncheberg, Germany; Thaer Institute, Faculty of Life Sciences, Humboldt University of Berlin, Invalidenstraße 42, 10115 Berlin, Germany

**Keywords:** consortium, Zn mobilization, Zn content, wheat, plant growth

## Abstract

Enhancing zinc (Zn) content in wheat grains by using Zn-mobilizing rhizosphere bacteria is becoming an eco-friendly and sustainable alternative to conventional approaches such as chemical fertilization. Our study aimed to isolate, screen, and evaluate Zn-mobilizing rhizosphere bacteria to improve its content in wheat biomass. Wheat rhizosphere soils were collected in several wheat planted soils in Ethiopia and Zn-solubilizing bacteria were isolated and screened for their plant beneficial traits. Isolates W8_A, W25_A, and W63_B were selected. Pot experiments were conducted in sterilized river sand supplemented with 0.05% zinc oxide by using these isolates. The experiment consisted of nine treatments in complete randomized design with four replications. Data were means of three independent experiments. W8_A, W25_A, and W63_B exhibited zinc solubilization index > 4.0. Consortium inoculation showed the highest improvements in plant growth. Inoculations improved shoot length and dry weigh on average by 67.4% and 84.2%, respectively compared to the control. On average, 90.2% root and 75.5% shoot Zn content increased compared to the untreated control. Hence, the isolates can be applied for Zn bio-fortification in wheat to combat Zn deficiencies in food grains.

## Teaser

Zn mobilizing rhizosphere bacteria: ecofriendly and sustainable alternatives to chemical fertilizers.

## Introduction

Zinc (Zn) is an essential micronutrient needed for the proper functioning of physiological and metabolic activities in all organisms (Natasha et al. 2022, Ali et al. 2023). It plays catalytic, co-catalytic, or structural functions in more than 300 enzymes (Hafeez et al. 2013). In addition, it has key role in carbohydrate and auxin metabolism in plants (Alloway 2008a,b), assimilation of nutrients and protein synthesis (Singh et al. 2018). Its deficiency causes reduction in photosynthesis, flowering and fruit development, lowers synthesis of carbohydrates, production of phytohormones, and growth and development of roots and shoot in plants (Shaikh and Saraf 2017). These lead to reduced water and nutrient uptake and delay in crop manurity and eventually leading to a decrease in crop yield and nutritional quality of food grains (Tavallali et al. 2010). Similarily, its deficiency affects billions of people across the globe, particularly in tropical and subtropical regions by causing stunted physical growth and retarded mental development in children (Shaikh and Saraf 2017).

Acidic or calcareous soils (common in the tropics) bind Zn or induce deficiency. Flooded paddy soils or rice fields (common in Asia) make Zn insoluble and thus not available for plants (Fischer et al. 2009). Roughly 75% of soils in sub-Saharan Africa and about 50% of cultivated Indian soils exhibit very low Zn contents (Fischer et al. 2009). Staple food crops such as rice, wheat and maize grown on these soils; therefore, have low grain Zn unless expensive Zn chemical fertilizers are applied (Nielsen 2012). Hence, the soil Zn deficiency directly limits the Zn content of common foods in tropical regions, because most smallholder farmers in low-income countries cannot afford Zn fertilizer (Fischer et al. 2009, Nielsen 2012, Hacisalihoglu 2020).

Zn deficiency in humans is a consquence of consumption of Zn deficient food grains including wheat grown in Zn deficient soils across the globe (Shaikh and Saraf 2017). However, soils have high total Zn content which exists mostly in a fixed and complex forms and un-available for plant use in the soil solution (Ali et al. 2023). Therfore, the widespread occurrenece of Zn deficiency in crops and foods across the globe is not soley due to the low level of Zn in the soil, rather it is because of low solubility of complex/fixed zinc compounds in the soil (Iqbal et al. 2010, Shaikh and Saraf 2017). In addition to this, Zn fertilizers applied into agricultural soils in the form of Zn sulphtates and Zn-EDTA (Anuradha et al. 2015) are ineffective, since 96%–99% of them is transformed into un-available Zn pools such as zinc carbonates, zinc oxides, zinc phosphates, zinc sulfides, zinc chlorides, etc by precipitation (Zhang et al. 2017, Ali et al. 2023).

To alleviate Zn deficiency and improve its bioavailability for plant nutrition and improve its concentration in food grains, several alternative approaches have been tried. Among others, the application of plant growth promoting rhizobacteria (PGPR) possessing Zn solubilizing capabilities became the most promising approach because it is cost effective, eco-friendly, sustainable, and from the soil to the soil alternative to improve Zn content in food grains (Yadav et al. 2022). Zn solubilizing bacteria indvidually, in consortium or in combination with organic materials enhanced bioavailability of native Zn in the soil (Yadav et al. 2022). Zn-solubilizing rhizosphere bacteria colonize the rhizosphere of plants and enhance Zn bioavailability by mobilizing insoluble and complex Zn compounds, and thereby improve plant growth, development, and yield. Mechanisms by which Zn-solubilizing rhizosphere bacteria mobilize compex and fixed Zn compounds include; production of organic acids and acidification of the rhizosphere soil thereby increase the solubility of complex and fixed Zn compounds (Masood et al. 2022, Yadav et al. 2022, Ali et al. 2023), proton extrusion and production of chelating ligands (Nain et al. 2010, Masood et al. 2022, Ali et al. 2023), production of inorganic acids such as nitric, sulphuric and carbonic acids which could also facilitate Zn solubilization in the rhizosphere soil (Nain et al. 2010).

Wheat (*Triticum aestivum* L.) is the most dominant staple food for billions of people across the globe. It covers more than 50% of diet in many micronutrient deficient regions in the world (Shaikh and Saraf 2017). However, wheat has inherently too low Zn content to meet the recommended dietary demand (generally 20–30 mg kg^-1^ of wheat grain) for human health (Cakmak 2008). Therefore, PGPR based bio-fortification of Zn in food grains such as wheat became most promising and sustainable alternative.

Wheat (*T. aestivum*) is the second most dominant staple food crop in Ethiopia, next to teff (*Eragrostis tef*). To the best of our knowledge, there is little or no information regarding the isolation, screening, and application of Zn-solubilizing rhizosphere bacteria from Ethiopian soils. We initiated this study to evaluate the contributions of Zn-solubilizing rhizosphere bacteria in enhancing wheat plant growth and improving Zn content in its biomass. Therefore, the objective of this study was to isolate, screen, and evaluate the Zn-solubilizing rhizosphere bacteria in enhancing wheat plant growth and improving root and shoot Zn content under greenhouse conditions.

## Materials and methods

### Soil sampling locations

**Figure 1:**
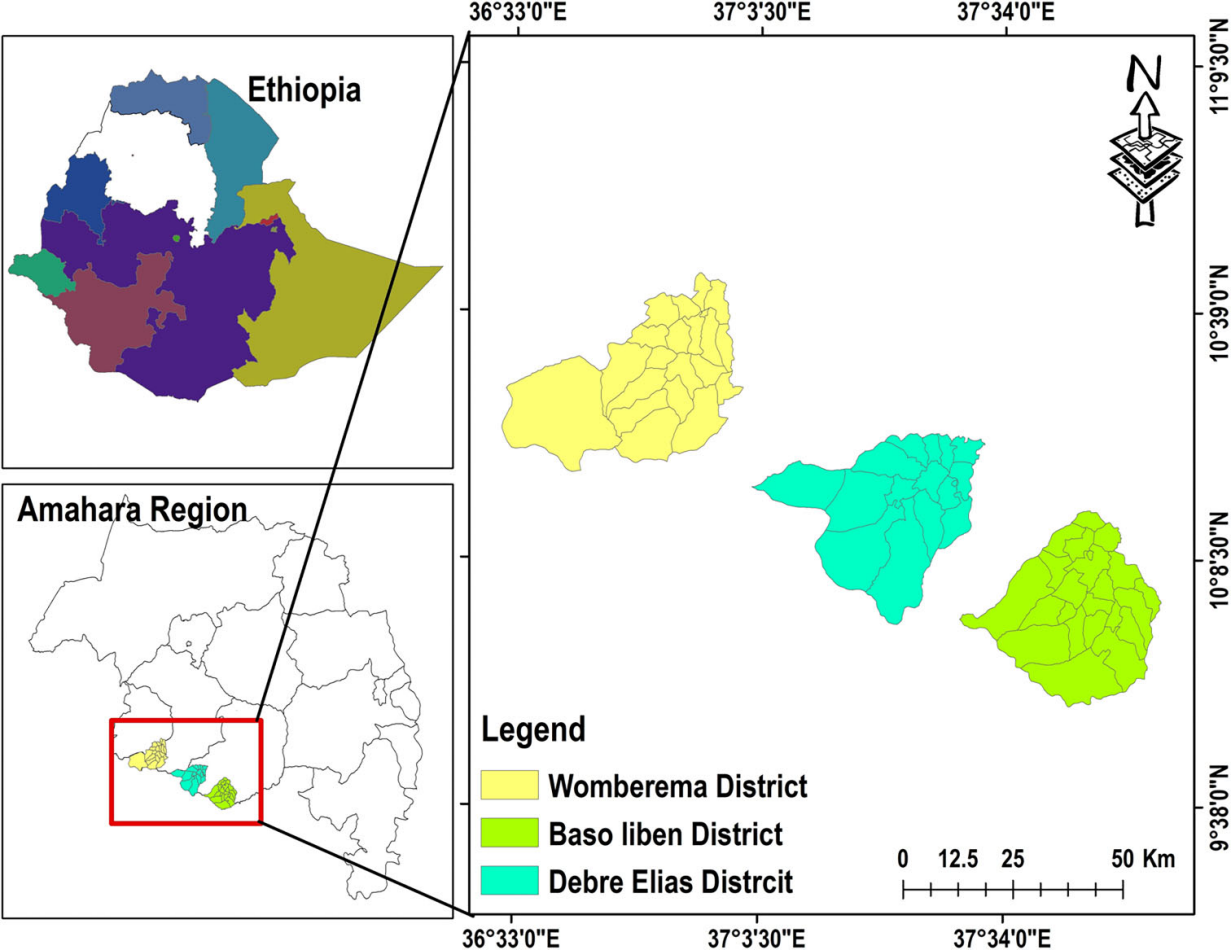
Map of the study areas.

The wheat rhizosphere soil samples were collected from three geographically separate locations namely; Debre Elias, Womberma and Baso-liben districts, Amhara National Regional State, Northern Ethiopia (Fig. 1). These districts were selected due to their higher wheat production in the region.

### Soil sampling, transporting, and storage

The sampling farms were randomly selected from each district. Five sampling points and wheat plants were identified in each farm. This was done by pointing out the four corners and the center of a rectangular farmland. Then, wheat plants were labeled and uprooted by digging 0–20 cm depth. The rhizosphere soils from the five sampling points were pooled into one composite sample and collected in sterile polyethylene bags. Totally, 32 soil samples (11 from Debre Elias, 11 from Womberma, and 10 from Baso-liben,) were collected for this study. Each pooled soil sample was about 1 kg and each sample was sub-divided into two portions: 500 g for Zn solubilizing rhizosphere bacteria isolation and the remaining 500 g was for soil physicochemical parameters analysis. The soil samples were transported to the Microbiology Laboratory, Department of Biology, Debre Markos University. The soil samples were stored at 4°C until further processing. The soil sampling was carried out from September—December 2020 cropping season. These months are times of wheat crop maturation and flowering at the study areas. The flowering stage marks a key physiological transition when the root activity and plant-microbe interaction are intense.

### Soil physicochemical analysis

The soil pH (pH-water) was determined according to the methods described in Ziadin and Tran (Ziadin and Tran 2007). Available phosphorous (P) was determined using the methods described in Olsen et al. (1954), total nitrogen (TN) was determined according to the Kjeldahl Method (Hinds 1980), the soil organic carbon was determined by using the methods described in Walkely and Black (1934), and soil potassium (K) was determined using Ammonium Acetate Method (Lewis et al. 1949). The soil micronutrients: Cu, Fe, Mn, and Zn were determined according to the DTPA Extraction method (Lindsay and Norvell 1978).

### Isolation and screening of Zn solubilizing rhizosphere bacteria

Zn-solubilizing rhizosphere bacteria isolation was done using tris-minimal salt medium (Fasim et al. 2002) and nutrient agar medium supplemented with 5.0 g of D-glucose (Costeroosse et al. 2017). The composition of tris-minimal salt medium was (g/l): Tris-HCl,6.6; NaCl,4.68; KCl,1.49; NH_4_Cl,1.07; Na_2_SO_4_, 0.43; MgCl_2_.2H_2_O, 0.2; CaCl_2_.2H_2_O, 0.03; D-glucose, 10 and Agar-agar, 15.0. In both media ZnO (0.629 g) and ZnCO_3_ (0.829 g) were separately added as sole sources of Zn (Costeroosse et al. 2017). The pH of the both media was adjusted to 7.0 and 50 mg/l cycloheximide was supplemented into each medium as an antifungal agent. The soil samples were manually crushed by hand and sieved using 2 mm size sieves. Then, 10 g of soil sample was serially diluted in 90 ml distilled sterile water. A 0.1 ml of appropriate dilution was spread plated on sterile pre-prepared tris-minimal salt medium and nutrient agar medium supplemented with D-glucose. The inoculated plates were incubated at 28±2°C for 7–10 days. Rhizosphere bacteria exhibiting halo clear zones around their colonies are considered as Zn-solubilizing rhizosphere bacteria and they were sub-cultured, purified, and stored at 4°C for further studies. Isolate designated by starting with “W” referring to wheat rhizosphere, followed by numbers to differentiate among isolates. Zn-solubilizing rhizosphere bacteria isolates were further screened and evaluated for the other insoluble Zn sources namely Zn_3_(PO_4_)_2_.2H_2_O and ZnCl_2_ by the spot inoculation method. For the spot inoculation study, 2 ml of 24–48 h old nutrient broth culture (10^8^ CFU/ml) was centrifuged at 10 000 r/m for 5 min. Then, the bacterial cell pellets were suspended in 50 µl distilled sterile water. A 20 µl of the bacterial cell suspension was spot inoculated on tris-minimal salt medium separately supplemented with zinc phosphate and zinc chloride and incubated at 28±2°C for 7–10 days. The Zn solubilization index (ZSI)for each isolate on respective media was determined by using the formula;


\begin{eqnarray*}
{\mathrm{ZSI}} = \frac{{{\mathrm{Halo\ zone\ diameter}} + {\mathrm{Colony\ diameter}}}}{{{\mathrm{Colony\ diameter}}}}.
\end{eqnarray*}

Isolates were ranked and selected for further characterization based on solubilizing diverse insoluble Zn sources and Zn solubilization index on each respective media.

### Quantitative estimation of Zn content by using broth assay

The quantitative estimation of the solubilized Zn by the selected rhizosphere bacteria isolates was done according to the methods described in Saravanan et al. (2004). The Zn-solubilizing rhizosphere bacterial isolates were inoculated separately to a basal medium supplemented with 0.1% zinc oxide. The basal medium was comprised of (g/l); glucose-10.0; ammonium sulphate-1.0; potassium chloride-0.2; dipotassium hydrogen phosphate-0.1; magnesium sulphate-0.2; and distilled water -1000 ml, pH 7.0. The solubilization of zinc from a laboratory grade ZnO (99.9%) by Zn solubilizing rhizosphere bacteria was estimated by adding 0.1% of zinc oxide into the basal medium. Then, the medium was prepared in 100 ml aliquots in 250 ml volume Erlenmeyer flasks and sterilized by autoclaving at 121°C for 30 min. Flasks were inoculated with 1 ml (10^8^ CFU/ml) of the test organism grown in nutrient broth and incubated at 28±2°C at a shaking speed of 200 r/m. The un-inoculated medium in the flasks was incubated together to serve as a control for each treatment. The experiments were carried out in triplicate. A 20 ml samples were withdrawn at the 3^rd^, 6^th^, and 9^th^ days after incubation and centrifuged at 10 000 r/m for 10 min to remove the debris and cells. The pH of each supernatant was determined using 10 ml supernatant and the remaining 10 ml of the supernatant was used for the determination of solubilized zinc content by using inductively coupled plasma-optical emission spectroscopy (ICP-OES) at the Leibniz Centre for Agricultural Landscape Research (ZALF), Germany.

### Evaluation of Zn-solubilizing isolates for multiple plant growth promoting traits

The potential candidate Zn-solubilizing rhizosphere bacteria isolates were evaluated for multiple plant growth promoting traits.

#### Phosphate solubilization

The potential candidate isolates were evaluated for inorganic phosphate solubilization by using Pikovskaya’s agar medium supplemented with tri-calcium phosphate (TCP) following the methods described in Berza et al. (2022b).The composition of the medium was (g/l): yeast extract, 0.5; dextrose, 10.0; tri-calcium phosphate, 5.0; ammonium sulphate, 0.5; potassium chloride, 0.2; magnesium sulphate, 0.1; manganese sulphate, 0.0001; ferrous sulphate, 0.0001; and agar–agar, 15.0. The pH of the medium was adjusted to 7.0 before autoclaving. Isolates were grown in a nutrient broth for 24–48 h at 28±2°C to exponential phase. A 2 ml of the bacterial culture (10^8^ CFU/ml) was centrifuged at 10 000 r/m for 5 min. The bacterial cell pellets were washed and suspended in 50 µl distilled sterile water and a 20 µl of this suspension was spot inoculated on the medium. Then, plates were incubated at 28±2°C for 7–10 days. Clear halo zones surrounding the bacteria colonies indicate phosphate solubilization. The phosphate solubilization index (PSI) was determined as follows:


\begin{eqnarray*}
PSI = \frac{{{\mathrm{\ colony\ diameter}} + {\mathrm{Halo\ zone\ diameter\ }}}}{{{\mathrm{Colony\ diameter}}}}.
\end{eqnarray*}

The quantitative estimation of phosphate solubilization by the isolates was done using National Botanical Research Institute’s phosphate (NBRIP) growth liquid medium (Nautiyal 1999). The isolates were separately grown for 24–48 h in a nutrient broth. A 30 µl broth cultures was inoculated into 50 ml NBRIP liquid medium in 100 ml volume flasks. The un-inoculated nutrient broths were included as a control. All the flasks (experimental and controls) were incubated at 28 ±2°C by shaking at 120 r/m for three consecutive days. A 10 ml culture was withdrawn and centrifuged at 13 000 r/m for 5 min and the supernatant was mixed with Barton’s reagent; (NH₄)_6_Mo_7_O₂₄·4H₂O was separately dissolved in water. NH₄VO₃ was also separately dissolved in boiling water then acidified in strong acid concentrated nitric acid (HNO₃)

The experiment was carried out in triplicates and repeated three times to authenticate the data. The released phosphate was determined by using colorimetric method (Berza et al. 2022a).

### IAA production

The isolates were grown in a nutrient broth for 24–48hat 28±2°C to exponential phase. The bacterial culture [2 ml (10^8^ CFU/ml)] was centrifuged at 10 000 r/m for 5 min and bacterial cell pellets were suspended in 50 µl distilled sterile water. A 20 µl of the bacterial cells pellet suspension was inoculated into a Luria Bertoni (LB) broth composed of (g/l): tryptone, 10; yeast extract, 5; NaCl, 2.5; and supplemented with L-tryptophan (0.1 g/l) as described in Gordon and Weber (1951). The un-inoculated LB broth medium was served as a control. The inoculated LB broth culture was incubated for 48 h at 28±2°C. About 3 ml of the LB broth culture was withdrawn and centrifuged at 10,000 r/m for 5 min and 2 ml of the supernatant was mixed with 2 ml Salkowski’s reagent (Gordon and Weber 1951). The composition of Salkowski’s reagent is (g/l): 50 ml, 35% per chloric acid (HClO_4_) and 1 ml of 0.5 M iron tri chloride (FeCl_3_). Then, the content was incubated in dark place at room temperature for 30 min. The appearance of pink coloration indicates IAA production, while non-production is indicated by the absence of color change. The intensity of pink coloration was rated as (+++) for deep pink coloration, (++) for pink coloration, (+) for pale red coloration and (−) for no color change. The colorimetric quantification of IAA was carried out by measuring the absorbance of the resulting solution at 535 nm using spectrophotometer (Berza et al. 2021).The experiment was done in triplicates and repeated twice to confirm the results.

### Siderophore production and ACC deaminase activity

The siderophore production assay was carried out according to the methods described in Othman et al. (2022). Briefly, the ability of our isolates to produce siderophore was assessed using Chrome Azurol S (CAS) agar plates. A fresh 20 µl cell pellet suspension (10^8^ cells/ml) of each bacteria isolate from LB broth was spot inoculated on the CAS agar media. The control CAS agar plates were inoculated with a sterile LB broth. Then, plates were incubated for 3–5 days at 28±2°C and the formation of an orange halo zone around the bacteria colonies indicate a positive results, as the siderophore is removed the Fe from the Fe-CAS dye complex in the CAS media (Yasmin 2021 et al. 2021). Determination of ACC deaminase activity of these rhizosphere bacteria isolates was carried out according to the methods and procedures described by Gupta and Pandey (2019). Briefly, these bacteria isolates were evaluated for their ACC deaminase activities on the sterile minimal DF (Dworkin and Foster) salt media, consisted of DF salts per litre: 4.0 g KH_2_PO_4_, 6.0 g Na_2_HPO_4_,2.0 g MgSO_4_.7H_2_O, 2.0 g glucose, 2.0 g, gluconic acid and 2.0 g citric acid with trace elements:1.0 mg FeSO_4_.7H_2_O, 10.0 mg H_3_BO_3_, 11.19 mg MnSO_4_.H_2_O, 124.6 mg ZnSO_4_.7H_2_O, 78.22 mg CuSO_4_.5H_2_O, 10.0 mg MoO_3_, pH 7.2 and finally amended with 3 mM ACC instead of (NH_4_)_2_SO_4_ as sole nitrogen source (Dworkin and Foster 1958). The controls plates were prepared by including (NH_4_)_2_SO_4_ (positive control) and the negative control plates were prepared without both the ACC and (NH_4_)_2_SO_4_. Plates were spot inoculated with a fresh 20 µl bacterial cell pellets suspension (10^8^ Cell/ml) and incubated for 5–6 days at 28±2°C. Bacteria growth was monitored on daily basis by comparing with both controls. The bacteria isolates which exhibited growth on the plates were considered ACC deaminase producer.

### Genomic DNA extraction and designation of phylogenetic position of the isolates

The bacterial total genomic DNA extraction was done according to the methods and procedures described in Goldenberger et al. (1995). Briefly, two loops bacterial colonies were homogenized in a 25 µl master mix containing ddH_2_O, 1 M NaOH, and 10% SDS. Then, the content was thoroughly vortexed and heat lysed at 95°C for 25 min. The quality and quantity of DNA was determined using a Nanodrop (Thermo Fisher Scientific). The partial 16S rRNA gene amplification was carried out by using primers 8F 5′-AGAGTTTGATCCTGGCTCAG-3′ and 1492R 5′-GGTTACCTTGTTACGACTT -3′. Sanger sequencing was carried out at LGC Genomics (Berlin, Germany). The partial 16S rRNA gene sequences of our isolates were deposited in the NCBI database under accession numbers PV449770, PV449771, and PV449772. The phylogenetic tree for the isolates was constructed by comparing the partial 16S rRNA gene sequences of the type strains obtained from the NCBI database. The ClustalW algorithm was used for sequence alignment and the Neighbor-Joining method was employed for phylogenetic tree construction with the bootstrap value of 1000 replications.

### Pot experiments to prove effectiveness of isolates on wheat

#### Experimental design and treatments

The greenhouse experiment was conducted at Debre Markos University, in 2022 and 2023. Plastic pots were surface sterilized by using 5% sodium hypochlorite solution. The pot size was 18 cm diameter and 25 cm height. Pots were filled with 3.5 kg sterile river sand which was thoroughly mixed with 0.05% (w/w) ZnO. The experiment was carried out in a completely randomized design and consisted of nine treatments with four replications. The isolates were selected based on their higher Zn solubilization index (ZSI) and the amount of solubilized and released Zn content in the liquid medium. The treatments were as follows:

T1-negative control consisted of ZnO, but without bacterial inoculation; T2- positive control (ZnSO_4_); T3-ZnO + W25_A; T4-ZnO + W8_A; T5-ZnO + W63_B; T6-ZnO + W25_A + W8_A; T7-ZnO + W25_A + W63_B; T8-ZnO + W8_A + W63_B; T9-ZnO + W25_A + W8_A + W63_B.

#### Seed preparation, bacterial inoculation, and plant biometric data collection

Wheat seeds (variety: Ogolcho) were obtained from the Ethiopian Institute of Agricultural Research (EIAR), Debre zeit Agricultural Research Centre (DZRC), Debre Zeit, Ethiopia. Wheat seeds were surface sterilized by using 3% sodium hypochlorite solution and germinated on sterile 1% (w/v) water agar. Three to five wheat seedlings were transplanted into each pot and thinned to two seedlings per pot after successful establishment. The compatibility among isolates was checked for co and consortia inoculations by cross striking method (Berza et al. 2022a). The bacterial culture of each isolate was grown in a nutrient broth for 24–48 h at 28 ±2°C. A 2 ml of bacterial culture (10^8^ CFU/ml) was inoculated per pot. Inoculation was done three times, at transplanting stage, 7 days after transplanting and 15 days after transplanting. Pots were regularly watered with distilled sterile water. The Hoagland plant growth nutrient solution was supplied 20 ml per pot on weekly basis for 40 days (Hoagland and Arnon 1950). The plant biometric data such as root and shoot length, root and shoot dry weight, root and shoot Zn content were determined. The root and shoot zinc content was determined using DTPA extraction method (Lindsay and Norvell 1978).

### Statistical data analysis

The data analyses were conducted by using SAS software version 9.4. Statistical significance tests in plant growth parameters and Zn contents among treatments were done by using one way analysis of variance (ANOVA). The mean comparisons were done by using LSD at 5% confidence interval.

## Results

### Soil physicochemical analysis

The results revealed that the soils in the sampling locations were acidic (Table 1). The pH of soils varied between 5.56 at Debre Elias district and 5.95 at Baso-Liben district. The highest available phosphorous (108.78 mg/kg), organic carbon (2.69%), and total nitrogen (0.22%) were recorded at Baso-Liben, Debre Elias, and Debre Elias districts, respectively. Similarly, the soil DTPA extractable Zn content also varied among sampling locations. Its range was between 0.37 mg/kg at Womberma district and 1.93 mg/kg at Baso-Liben district (Table 1).

**Table 1:** Physicochemical characteristics of soil from the sampling districts.

	Districts		
Soil parameters	Baso liben	Womberma	Debre Elias
pH (H_2_O)	5.95	5.82	5.56
P (mg/kg)	108.78	15.57	18.17
K (mg/kg)	8.23	0.67	2.14
Fe (mg/kg)	16.36	52.92	31.94
Mn (mg/kg)	6.33	38.50	113.00
Zn (mg/kg)	1.93	0.37	1.08
Cu (mg/kg)	0.38	3.68	3.56
OC (%)	1.15	1.60	2.69
TN (%)	0.11	0.14	0.22

### Isolation and screening Zn-solubilizing rhizosphere bacteria

**Figure 2:**
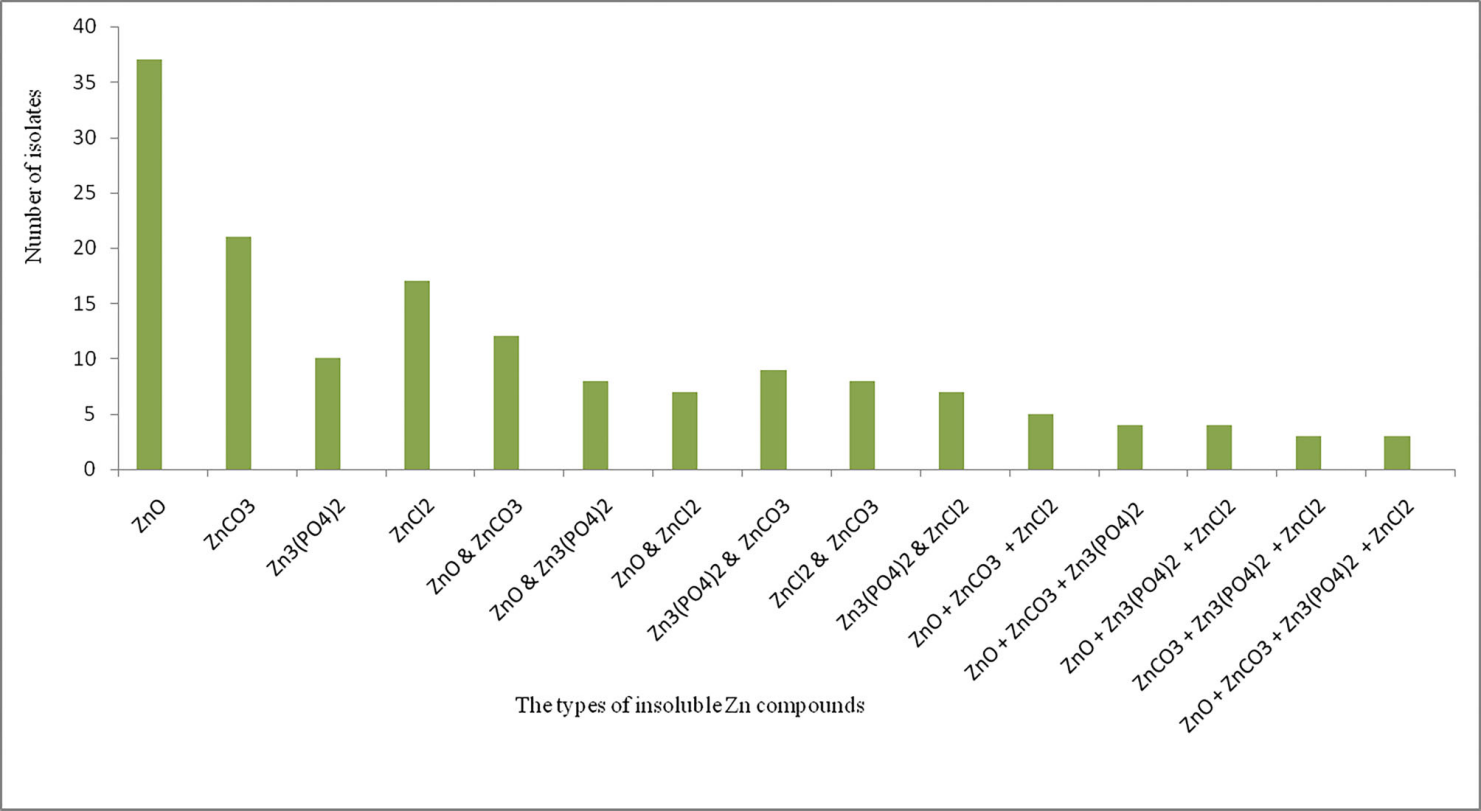
The number of Zn-mobilizing rhizosphere bacteria able to solubilize different insoluble Zn compounds. Tris-minimal salt medium was supplemented with 0.629 gram, 0.829 gram, 0.500 gram, and 0.4 gram of Zn-oxide, Zn-carbonate, Zn-phosphate and Zn-chloride, respectively in 1000 ml.

In this study, we isolated a total of 54 zinc solubilizing bacteria from the rhizosphere of wheat from the three districts (Supplementary Table 1). Among 54 isolates, 37 (68.5%) were exhibited ZnO solubilization, while the remaining exhibited ZnCO_3_ solubilizing abilities. In addition, 17 (31.5%) solubilized ZnCl_2_ solubilizing abilities, while 10 (18.5%) solubilized zinc phosphate. Among 54 isolates, only 3(5.5%), solubilized ZnO, ZnCO_3_, ZnCl_2_, and ZnPO_4_ (Fig. 2).

**Figure 3:**
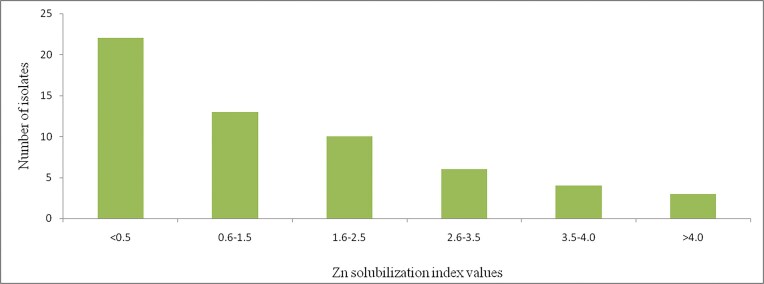
The number of Zn solubilizing rhizosphere bacteria and Zn solubilization index on zinc oxide. Tris-minimal salt medium was supplemented with 0.629 gram of Zn-oxide in 1000 ml.

These rhizosphere bacteria isolates exhibited variable Zn-solubilizing potentials on tris-minimal salt medium supplemented with ZnO as revealed by Zn solubilization index (ZSI) (Fig. 3). Some of the isolates 22 (40.7%) exhibited ZSI below 0.5, whereas 13 (24.1%) of the isolates showed ZSI between 0.5 and 1.5 (Fig. 3). However, 7 (12.9%) of the isolates exhibited zinc solubilization index greater than 3.5. Among the seven higher Zn solubilizers, only three isolates exhibited Zn solubilization index greater than 4.0. These potential isolates were designated as W25_A, W8_A, and W63_B. They exhibited abilities of solubilizing insoluble zinc compounds, zinc oxide, zinc carbonate, zinc phosphate, and zinc chloride apart from producing zinc solubilization index greater than 4.0 (Fig. 3).

**Figure 4:**
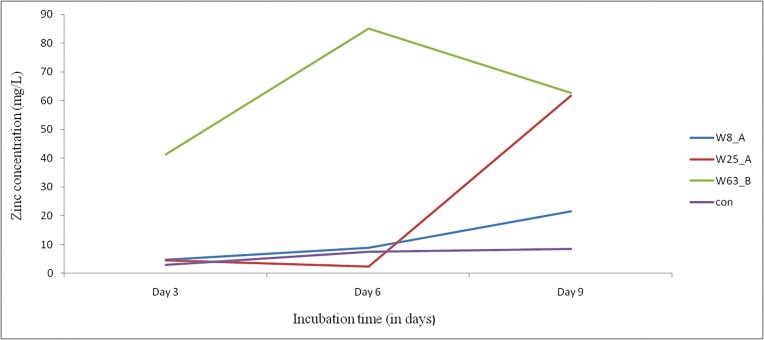
The amount of solubilized Zn by the potential rhizobacteria isolates in liquid medium at the incubation periods.

The quantitative determination of solubilized Zn content in liquid medium is presented in Fig. 4. The highest mean solubilized Zn content (41.36 mg/l) was recorded by W63_B followed by 4.80 mg/l by W8_A at day 3. At day 6, the highest mean solubilized Zn content (85.16 mg/l) was recorded by W63_B. Similarly, the highest mean solubilized Zn contents, 62.82, 61.63, and 21.56 mg/l were recorded by W63_B, W25_A, and W8_A, respectively at day 9 (Fig. 4).

### Evaluation of Zn-solubilizing isolates for multiple plant growth promoting traits

The isolates exhibited variable phosphate solubilization, siderophore production, ACC deaminase activity, and IAA production capabilities (Table 2). All the three isolates were phosphate solubilizers and exhibited phosphate solubilization efficiencies with PSI values varying between 1.2 and 4.1. Isolate W63_B exhibited the highest mean phosphate solubilization index (4.1) and 68.5 µg/ml solubilized and released phosphates followed by 2.7 PSI and 32.1 µg/ml solubilized phosphates which were due to the inoculation of W8_A. Similarly, the highest mean IAA (23.5 µg/ml) was recorded by W25_A followed by 11.7 µg/ml IAA produced by isolate W63_B (Table 2). In addition, all the three isolates exhibited siderophore production, and isolates W8_A and W63_B further exhibited ACC deaminase activity (Table 2).

**Table 2:** The plant growth promoting traits of zinc solubilizing rhizobacteria.

Isolates	Phosphate solubilization	IAA production	Siderophore production	ACC deaminase production
W25_A	(1.2) 8.2 µg/ml	+++, 23.5 µg/ml)	+	-
W8_A	(2.7) 32.1 µg/ml	+, 6.4 µg/ml)	+	+
W63_B	(4.1) 68.5 µg/ml	++, 11.7 µg/ml)	+	+

Phosphate solubilization; numbers in the parentheses indicate phosphate solubilization index, IAA production; +++ - deep pink coloration, ++ - pink coloration, + - pale red coloration; Siderophore and ACC deaminase production; + -positive, -negative.

#### Evaluation of plant growth promoting traits under Zn-deficient greenhouse condition

We also assessed the isolates; W25_A, W8_A, and W63_B for insoluble zinc solubilization and plant growth promoting traits in the Zn deficient sterile river sand under greenhouse conditions. Results from compatibility study revealed that all our isolates didn’t show antagonistic behavior among one another (Supplementary Fig. 1). Different treatments exhibited significant (*P* < 0.05) differences in wheat plant root length. All the treatments consisted of Zn solubilizing rhizosphere bacteria shown improvements in root length compared to the negative control (Table 3). The highest root lengths (48.13 cm) and (46.22 cm) were recorded by the consortium inoculation (T-9) and dual inoculation consisted of W8_A and W63_B (T-8), respectively. However, the smallest root lengths (24.38 cm) and (35.75 cm) were recorded by the negative (T-1) and positive control treatments (T-2), respectively (Table 3). Bacteria inoculations improved root length by 74.4% on average compared to the negative control. The smallest root length improvement (68.7%) was recorded in treatments T-3 and T5, while the highest improvement (97.4%) was exhibited by consortium inoculation (T-9) compared to the negative control. Similarly, we observed significant (*P* < 0.05) differences in plant shoot lengths among inoculation treatments. In this context, the highest shoot lengths (51.25 cm) and (45.88 cm) were recorded by the bacteria inoculation treatments consisted of the consortium (T-9) and co-inoculation treatment (T-8), respectively (Table 3). The smallest shoot lengths (26.13 cm) and (39.13 cm) were recorded by the negative control plants (T-1) and the positive control plants (T-2), respectively. Similar to the roots, bacteria inoculations improved plant shoot length at least by 50.2% (T-5) and at most by 96.1% (T-9) compared to the control. The average shoot length improvement in this study was 67.4% compared to the negative control.

**Table 3:** Wheat plant growth parameters after inoculation with Zn solubilizing rhizobacteria in sand culture. Data are recorded after 40 days growth under greenhouse conditions.

Treatment	Code	RL(cm)	SHL(cm)	RDWT(g)	SHDWT(g)
Negative control	T1	24.38±0.9e	26.13±0.1d	0.35±0.00d	0.34±0.0d
Positive control	T2	35.75±0.5d	39.13±0.8c	0.63±0.03b	0.71±0.1b
ZnO+W25_A	T3	41.13±0.2c	43.38±0.3cb	0.44±0.01c	0.41±0.2c
ZnO+W8_A	T4	41.25±0.8c	42.25±0.6cb	0.46±0.02c	0.44±0.0c
ZnO+W63_B	T5	41.13±0.1c	39.25±0.0c	0.44±0.03c	0.39±0.1dc
ZnO+W25_A+W8_A	T6	45.25±1.0bac	44.81±0.9b	0.65±0.02b	0.74±0.3b
ZnO+W25_A+W63_B	T7	42.00±1.0bc	43.64±0.7cb	0.68±0.01b	0.74±0.0b
ZnO+W8_A+W63_B	T8	46.22±1.0ba	45.88±1.1b	0.64±0.0b	0.72±0.2b
ZnO+W25_A+W8_A+W63_B	T9	48.13±1.1a	51.25±1.2a	0.76±0.09a	0.86±0.1a

RL = root length, SHL = shoot length, RDWT = root dry weight and SHDWT = shoot dry weight. Values are mean ±SD and are expressed as means of triplicate experiments. Means with the same letter in the same column are not significantly different at *P* < 0.05 by LSD test.

In addition, significant (*P* < 0.05) differences in root and shoot dry weight of wheat plants were recorded among treatments. In this context, the highest root dry weights (0.76 g) and (0.68 g) were recorded in the treatments consisted of consortium inoculation (T-9) and by the co-inoculation treatment comprised of W25_A + W63_B (T-7), respectively (Table 3). Similarly, the highest shoot dry weight (0.86 g) was recorded by the treatment that consisted of consortium of the three isolates (T-9), followed by (0.74 g), which was recorded by the treatment consisted of co-inoculation of W25_A + W8_A (T-6), and the co-inoculation treatment consisted of W25_A+ W63_B (T-7). However, the smallest shoot dry weights (0.34 g) and (0.41 g) were recorded by the negative control (T-1) and by the single inoculation treatment consisted of W25_A (T-3), respectively. All bacteria inoculations improved root and shoot dry biomass compared to the negative control. The highest root dry weight (117.1%) and the highest shoot dry weight (152.9%) improvements were recorded by consortium inoculation treatments (T-9) compared to the negative control. On average, in this study, about 67.8% root dry weight and 84.2% shoot dry weight improvements were recorded compared to the negative control.

### Zinc content of the wheat plant biomass under the greenhouse conditions

**Figure 5:**
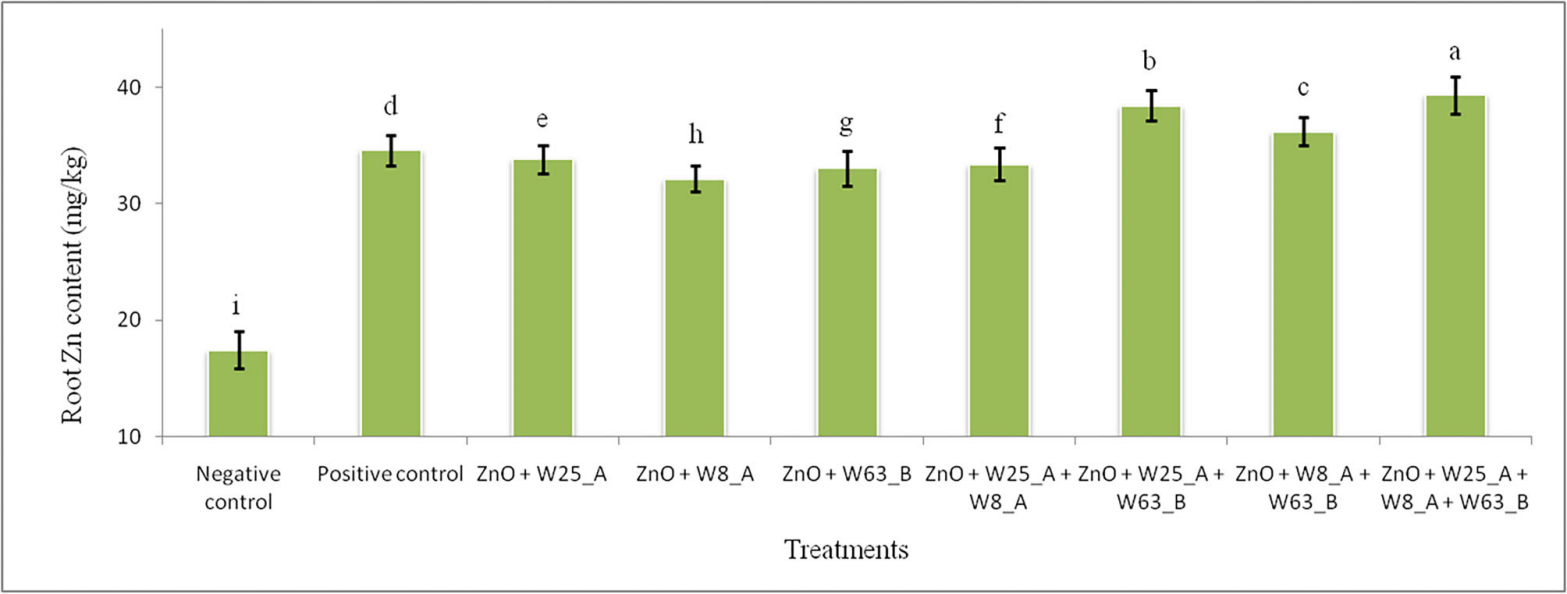
Effects of inoculation of wheat plants with Zn solubilizing rhizosphere bacteria on the root Zn content in sand culture under greenhouse conditions.

Isolates W25_A, W8_A, and W63_B individually and/or in combination were evaluated for their potential in improving wheat plant biomass Zn content (root and shoot), when grown in Zn deficient-soil free growth settings under greenhouse conditions. In this context, we recorded significant (*P* < 0.05) differences in root and shoot Zn content among treatments. The highest root Zn contents, 39.35 mg/kg and 38.41 mg/kg were recorded in the treatments consisted of the consortium of W25_A + W8_A + W63_B (T-9) and dual inoculation treatment comprised of W25_A + W63_B, respectively (Fig. 5). However, the smallest root Zn contents, 17.43 mg/kg and 32.15 mg/kg were recorded in the negative control (T-1) and in the single inoculation with W8_A (T-4), respectively. All bacteria inoculated treatments exhibited root Zn content improvement compared to the negative control treatment. The highest (125.7%) root Zn content improvement was exhibited by the consortium inoculation (T-9) compared to the negative control, while the smallest (84.4%) root Zn content improvement was recorded by T-4. Inoculation of rhizosphere bacteria improved root Zn content by 90.2% on average compared to the negative control. It is very interesting to observe that the co- and consortium inoculations improved root Zn content even better than the positive control. In this context, the dual inoculation treatments, T-7, T-8, and consortium inoculation (T-9) improved the root Zn content by 11.1%, 4.7%, and 13.8%, respectively compared to the positive control (T-2).

**Figure 6:**
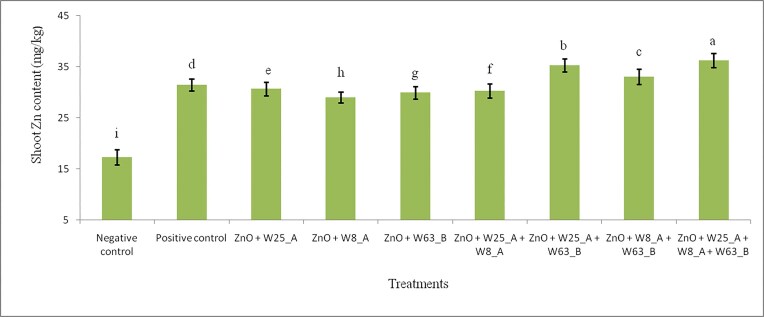
Effects of inoculation of wheat plants with Zn solubilizing rhizosphere bacteria on the shoot Zn content in sand culture under greenhouse conditions.

In addition, we recorded a significant (*P* < 0.05) differences in shoot Zn content among treatments. The highest shoot Zn content (36.22 mg/kg) was recorded by the consortium inoculation (T-9), followed by 35.28 mg/kg recorded by the dual inoculation of W25_A + W63_B (T-7) (Fig. 6). But, the smallest shoot Zn content (17.30 mg/kg) was recorded by the negative control (T-1), followed by 29.02 mg/kg due to a single inoculation with W8_A (T-4). In general, rhizosphere bacteria inoculations improved shoot Zn content between 67.7% and 109.4% compared to the negative control. In particular, the highest (109.4%) shoot Zn content improvement was recorded by the consortium inoculation (T-9) and the average shoot Zn content improvement was about 75.5% compared to the negative control. Moreover, dual inoculations T-7, T-8 and the consortium (T-9) improved shoot Zn content better than the positive control. That is, treatments, T-7, T-8, and T-9 improved the shoot Zn content by 12.2%, 5.2%, and 15.2%, respectively, compared to the positive control.

### The phylogenetic positioning of isolates

**Figure 7:**
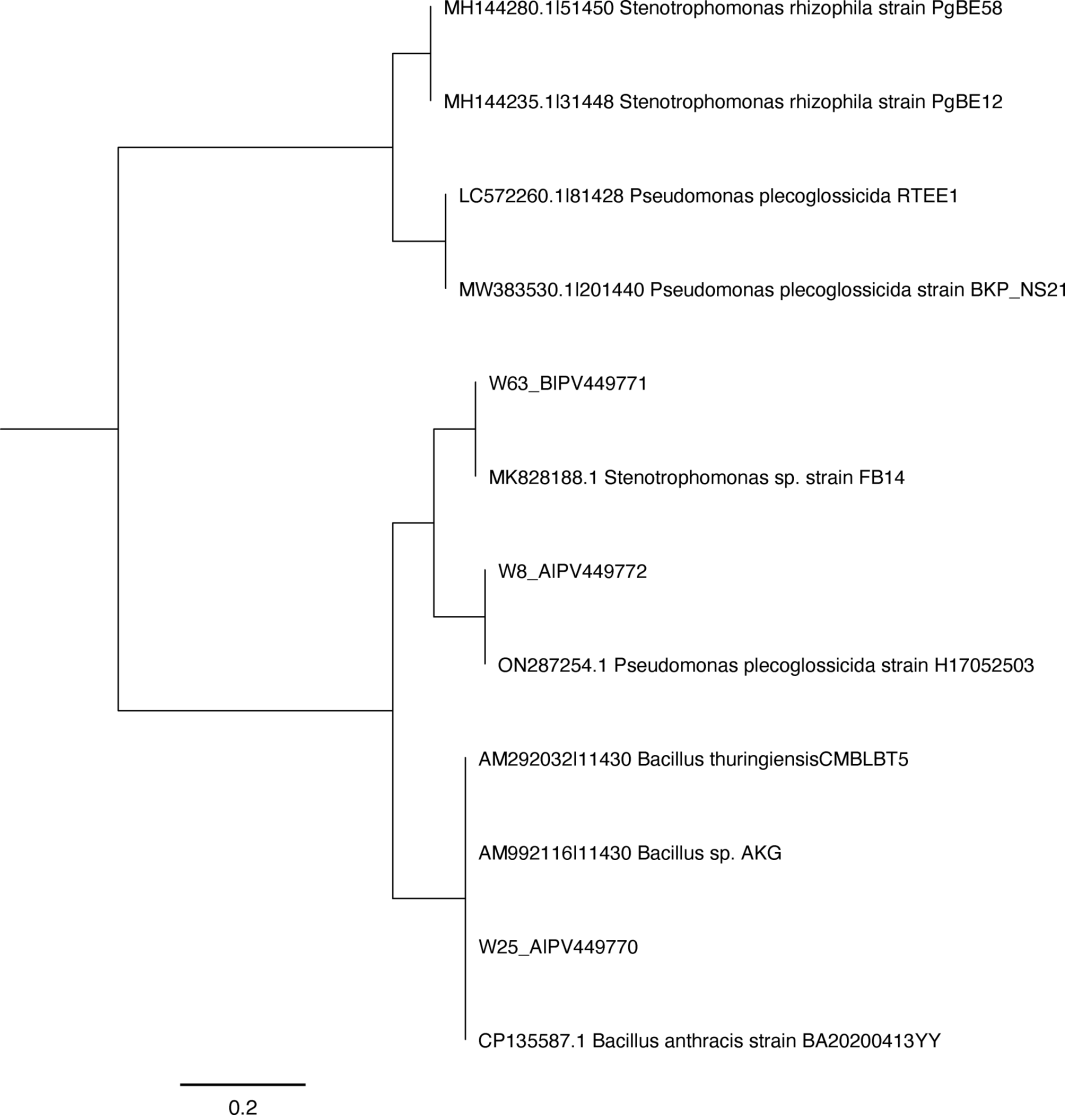
Phylogenetic tree constructed using partial 16S rRNA gene sequences of the potential isolates. Bootstrap values are calculated from 1000 replications using neighbor-joining method.

The partial 16S rRNA gene analysis has placed these rhizosphere bacteria isolates into their phylogenetic positions. The 16S rRNA gene sequence similarity of our rhizosphere bacteria isolates and the reference strains from National Center for Biotechnology Information (NCBI) database is presented in Table 4. The partial 16S rRNA gene sequence analysis has identified and grouped our isolates into three distinct categories. Based on the sequence identity, isolates W8_A and W63_B are affiliated to Proteobacteria, particularly to Gamma proteobacteria, whereas isolate W25_A is affiliated to Bacillota and belongs to the Bacillus. Isolate W8_A is identified to be *Pseudomonas plecoglossicida*, Isolate W25_A belongs to *Bacillus paramycoides* and W63_B is to *Stenotrophomonas rhizophila* (Table 4: Fig. 7). These bacteria species are the first reports to the science from the Ethiopian soils, particularly from wheat rhizosphere. Similarly, this work is the first report to the science regarding zinc mobilization by these bacteria species.

**Table 4:** Identity of the isolates based on partial 16S rRNA gene sequences analysis.

Strain	Species name	Accession number	Query length (bp)	Best match ID	Similarity
W8_A	*Pseudomonas plecoglossicida*	PV449772	1442	LMG 16121	99.93
W25_A	*Bacillus paramycoides*	PV449770	1451	W14	99.93
W63_B	*Stenotrophomonas rhizophila*	PV449771	1467	NBRC 14164	99.93

## Discussion

### Soil nutrients and, isolation, screening, and Zn solubilization efficiency

It is general truth that the majority of plant nutrients (macro and micro) which are available in the plant biomass come from the soil solution. Therefore, understanding the status of soil nutrients and the factors affecting their availability is very crucial in research. In this context, soils in our sampling locations were acidic and their DTPA extractable Zn content was highly varied among districts (Table 1). The main soil factors which affect the availability of Zn to the plants in the soil and control the amount of Zn in the soil solution include: total Zn and clay content, redox potential, activity of soil microorganisms, presence of other nutrients, and climatic conditions (Noulas et al. 2018, Van Eynde et al. 2023). Besides, the bioavailable Zn fractions in the soil include: its soluble, exchangeable, and complexed forms and studies showed that DTPA extractable Zn is correlated well with plant uptake (Long et al. 2003).

Our isolates exhibited varied Zn solubilization capabilities for the four mineral Zn compounds used in this study. We recorded ZSI values between 0.3 and 4.5. Most of the isolates solubilized ZnO followed by ZnCO_3_. Similar zinc solubilization index values were recorded by rhizosphere bacteria between 1.5 and 3.8 (Saravanan et al. (The quantitative estimation of the solubilized Zn by the selected rhizosphere bacteria isolates was done according to the methods described in Saravanan et al. 2004), 2.63 and 4.2 (Ali et al. 2023), and 2.50 and 3.72 (Bagmare and Ismail 2023) on plate assay supplemented with ZnO. However, a much higher ZSI value, between 8.22 and 11.37 was recorded by rhizosphere bacteria on zinc phosphate supplemented plate assay (Yadav et al. 2022). Variations in the degree of Zn-solubilization by rhizosphere bacteria have been reported by several scholars. Similar to our results, Saravanan et al. (2004) have also reported a higher Zn solubilization index on zinc oxide supplemented medium compared to the other media supplemented with the other Zn compounds. These variations could be due to differences in Zn solubilizing capabilities of the isolates and also their abilities to adhere to the insoluble metal compounds (Saravanan et al. (The quantitative estimation of the solubilized Zn by the selected rhizosphere bacteria isolates was done according to the methods described in Saravanan et al. 2004, Shaikh and Saraf 2017). Apart from Zn mobilizing ability, our isolates were endowed with multiple plant beneficial traits, such as phosphate solubilization, IAA and siderophore production and ACC deaminase activity. Phosphate solubilizing rhizosphere bacteria assist plants by mobilizing and availing insoluble forms of phosphates and IAA improves root growth and development (Berza et al. 2022a, b). Siderophores produced by the rhizosphere bacteria bind to iron, making it unavailable to the pathogens and promoting plant growth. Such activities enhance iron uptake by plants, boost plant growth and help controlling plant diseases by limiting pathogens access to iron (Sultana et al. 2021). IAA may also help in inducing ACC synthase to increase ACC production, which is used by the ACC deaminase to decrease ethylene production (Carlos et al. 2016), thereby help plants to tolerate biotic and abiotic stressors.

### Zn mobilizing phyto-beneficial bacteria inoculation improves plant growth

In this study, significant improvements in plant growth and yield were recorded in wheat plants grown in Zn deficient sterile river sand supplemented with ZnO and inoculated with Zn solubilizing phyto-beneficial rhizosphere bacteria. All treatments inoculated with Zn solubilizing rhizosphere bacteria showed significant improvements in root and shoot lengths, and root and shoot dry weights compared to the negative control. Treatments consisted of zinc solubilizing bacteria exhibited on average 74.4%, 67.4%, 67.8%, and 84.2% improvement in root length, shoot length, root dry weight, and shoot dry weight, respectively compared to the negative control. Such a higher plant growth and yield improvements could be most probably associated to enhanced plant growth and development, thereby result in the improved plant nutrient uptake. This improvement could be due to the fact that our Zn solubilizing bacteria possess multiple plant growth promoting properties such as phosphate solubilization, sideophore production, ACC deaminase activities, and IAA production apart from Zn solubilization. The inoculation of Zn solubilizing bacteria might have accelerated bioavailability of Zn due to mineralization and solubilization its compounds, and provide plants with a sufficient amount of solubilized Zn (Kamran et al. 2017, Ali et al. 2023). Moreover, IAA production by the Zn solubilizing rhizosphere bacteria might have improved plant growth and biomass, which collectively led to the enhanced induction of plant physiological processes (Kamran et al. 2017, Shaikh and Saraf 2017). Ali et al. (2023) have reported significant improvements in plant growth and yield, due to inoculation with zinc solubilizing rhizosphere bacteria and zinc oxide in sand culture under greenhouse conditions. They recorded up to 116%, 10%, 435%, and 70% improvements in root length, shoot length, root dry weight, and shoot dry weight, respectively compared to the negative control. These authors have report much higher improvements in root related growth parameters, very small improvement in shoot length and comparable improvements in shoot dry weight compared to our work. These variations could be attributed to the differences in Zn solubilizing capabilities among rhizosphere bacteria, variations in seed varieties, differences in the plant growth period, growth conditions, and the amount of zinc oxide supplemented in the sand culture. Kamran et al (2017) have also recorded improvements in wheat plant growth and yield, as a result of inoculation with Zn solubilizing bacteria in sand culture supplemented with zinc carbonate. These authors have reported up to 33%, 6.8%, 98%, and 32% improvements in wheat plant root length, shoot length, root dry weight, and shoot dry weight, respectively. Kamran et al. (2017) have recorded less improvement in most growth parameters except root dry weight compared to our work. These variations could be due to differences in Zn solubilizing and plant growth promoting capabilities among rhizosphere bacteria, time length of growth (30 days in their case, while 40 days in our study), wheat seed variety/genetic difference and variation in growth conditions. In addition, Panda et al. (2023) have recorded higher plant growth enhancement due to single inoculation of rice plant with Zn solubilizing rhizosphere bacteria.

### Zn mobilizing rhizosphere bacteria inoculation improves plant biomass Zn content

Inoculations of wheat plant with Zn solubilizing rhizosphere bacteria in sand culture supplemented with zinc oxide has improved the root and shoot Zn content on average by 90.2% and 75.5%, respectively compared to the negative control. The plant biomass usually contains Zn concentration in the range of 10–100 mg/kg (Broadley et al. 2007, Wang et al. 2014), hence our results are in line with these scholars. Our results revealed that higher Zn contents were observed wheat plant roots compared to shoots in all treatments. The most probable reason for this could be the binding of Zn to opposite charged sites in the cell walls of plant roots and enhanced Zn storage in the vacuoles of the cell and resulted in reduced translocation to the shoots in the presence of higher Zn availability (Greger 2004). The other possible reason may be the transporting efficiency of Zincol-16 transporters of the wheat genotype. In this context, Imtiaz et al. (2017) have recorded efficient and inefficient wheat genotypes on the basis of Zn translocation. We also recorded Zn content in the negative control which contained zinc oxide but not inoculated with zinc solubilizing rhizosphere bacteria. The Zn content in the negative control plants could be came from Zn in the sand and/or the water soluble fractions of zinc oxide. In addition, dual and consortium inoculations exhibited better and higher Zn content improvements in root and shoot compared to the single inoculations. These improvements revealed that the inoculated rhizosphere bacteria significantly contributed to the enhancement of the bioavailability and translocation of Zn into roots and shoots, and thereby provided the wheat plants with more available nutrients. Yadav et al (2022) have reported a comparable result in root Zn content improvement in the range of 2.4%–86.7% from wheat plants grown in sand culture supplemented with zinc phosphate and inoculated individually and/or in combination with Zn solubilizing rhizosphere bacteria. However, Karmran et al. (2017) have recorded very higher improvements compared to our results in wheat plant root Zn content which was in the range of 595.2% and 1048.6% in wheat plants grown in sand culture containing zinc carbonate and inoculated with Zn solubilizing rhizosphere bacteria individually and/or in consortia form.

Similarly, Ali et al. (2023) have reported shoot Zn content improvement in the range of 3.3%–47.5% from a wheat plant grown in sand culture supplemented with zinc oxide and inoculated with Zn solubilizing rhizosphere bacteria individually and/or in combination. These are smaller compared to our results. However, Kamran et al. (2017) have recorded much higher shoot Zn content improvement in the range of 182.3%–329.4% in wheat plant grown in sand culture containing zinc carbonate inoculated with Zn solubilizing rhizosphere bacteria individually and/or in consortium. In addition, Yadav et al. (2022) have reported higher shoot Zn content improvements in the range of 7.1%–185.6% from wheat plants grown in sand culture supplemented with zinc phosphate and inoculated with Zn solubilizing rhizosphere bacteria individually and/or in consortia. These variations could arise most probably from differences Zn solubilizing ability of bacterial isolates, genotypic variation in wheat plants, and zinc sources applied in the experiments. The improvement could be due to the bioavailability of Zn to the plant up take as a result of mobilization from zinc oxide by zinc solubilizing rhizosphere bacteria. Moreover, Zn concentration improvement in the shoot and root of wheat plants due to the enhanced zinc oxide mobilization, nutrient absorption and translocation towards roots, shoots by the help of Zn solubilizing rhizosphere bacteria. The enhanced plant growth and development encourages enhanced translocation of available Zn, which in turn led to the enhanced induction of physiological processes (Lucas et al. 2014, Olanrewaju et al. 2017, Wei et al. 2022, Ali et al. 2023). However; if its concentration is greater than 300 mg/kg, then Zn toxicity becomes a problem and causes chlorosis (Broadley 2007 et al. 2007).

Several studies have shown that rhizosphere bacteria have been used in countering plant nutrient deficiency and improving plant health, when applied as bio-fertilizers and bio-control agents. In this context, Shaikh and Saraf (2017) have recorded enhanced nutrient uptake, plant growth and development, improved yield, enhanced immune system, and health of a plant, inoculated with plant growth promoting rhizobacteria. Similarly, inoculations of different cereal crops with Zn solubilizing rhizosphere bacteria increased mobilization of zinc and also boosted its translocation towards roots, hoot, and grains. In this study, because of the ability of our isolates to successfully execute the plant-microbe interactions such as induction of plant physiological processes; solubilization and mobilization of complex Zn, and other minerals as reported by Ramesh et al. (2014), Kamran et al. (2017), Shaikh and Saraf (2017), Yadav et al. (2022), and Ali et al. (2023). The major advantage of inoculating plant-beneficial rhizosphere bacteria in crop production is their dual beneficiary effects, serving as complete bio-fertilizers as well as their significant roles in the bio-fortification of wheat (Shaikh and Saraf 2017). The application of plant-beneficial microbes is a sustainable solution for resolving micronutrient deficiency and avoiding agrochemicals in crop production. Zn-mobilizing rhizosphere bacteria are one of the most promising biological technologies to simultaneously improve wheat yields (bio-fertilization) and increase grain zinc availability (bio-fortification). Zn-solubilizing rhizosphere bacteria can be applied to the soil via several modes including (1) seed treatment or seed coating, (2) soil or row inoculation during sowing, (3) co-application with reduced Zn fertilizers, (4) foliar sprays by using microbial metabolites, and (5) consortia (so called Sythetic communities, SynComs) and bio-fertilizer blends (Kamran et al. 2017, Upadhayay et al. 2022, Yadav et al. 2023, Malik et al. 2025).

## Conclusions

Inoculation of wheat plant with Zn-solubilizing rhizosphere Bacteria individually, bi-culture of two strains or as synthetic consortium (SynCom), enhanced wheat biomass, and its biomass Zn content. Most of dual strain and consortium inoculations improved wheat plant root and shoot length, and root and shoot dry weight even more than the positive control. Similarly, most of the dual strain and SynCom inoculations significantly improved wheat plants’ root, shoot, and grain Zn content. In addition, the consortium inoculation improved wheat root, shoot, and grain Zn content higher than even the positive control. The Zn-solubilizing rhizosphere bacterial isolates, W8, W25, and W63 can be applied for Zn bio-fortification in wheat and also for its bio-fertilization. Since this work is the first from Ethiopian soils that characterized by Zn inavailalibility, these soils seem to be potential reservoirs for Zn-solubilizing rhizosphere Bacteria prone to future exploration of their plant growth promoting potential beyond wheat cropping systems.

## Supplementary Material

fiag030_Supplemental_Files
