# Supplementary material for: Zn-mobilizing bacteria improve shoot biomass and zinc content in wheat
Source: FEMS Microbiol Ecol. 2026 Apr 13;102(5):fiag030. doi: 10.1093/femsec/fiag030 (PMC13089531; doi:10.1093/femsec/fiag030)
Supplement: fiag030_Supplemental_Files [file fiag030_supplemental_files.zip › Supplementary Table 1.docx]

Supplementary Table 1: List of Zn mobilizing wheat rhizosphere bacteria and their respective Zn solubilization index

| S/No | Isolate code | Zn solubilization index | Phosphate solubilization index | IAA production | Siderophore production | ACC deaminase activity |
| --- | --- | --- | --- | --- | --- | --- |
| 1 | W1 | 0.1 | ND | ND | ND | ND |
| 2 | W2 | 0.1 | ND | ND | ND | ND |
| 3 | W3 | 0.5 | ND | ND | ND | ND |
| 4 | W4 | 1.4 | ND | ND | ND | ND |
| 5 | W5 | 1.2 | ND | ND | ND | ND |
| 6 | W6 | 0.1 | ND | ND | ND | ND |
| 7 | W7_A | 2.9 | ND | ND | ND | ND |
| 8 | W7_B | 1.1 | ND | ND | ND | ND |
| 9 | W8_A | 4.12 | 2.7 | + | + | + |
| 10 | W9 | 2.6 | ND | ND | ND | ND |
| 11 | W10 | 0.4 | ND | ND | ND | ND |
| 12 | W11 | 2.5 | ND | ND | ND | ND |
| 13 | W12_A | 1.3 | ND | ND | ND | ND |
| 14 | W12_B | 0.2 | ND | ND | ND | ND |
| 15 | W13 | 0.1 | ND | ND | ND | ND |
| 16 | W14 | 1.4 | ND | ND | ND | ND |
| 17 | W15 | 3.8 | ND | ND | ND | ND |
| 18 | W16_A | 0.4 | ND | ND | ND | ND |
| 19 | W16_B | 3.2 | ND | ND | ND | ND |
| 20 | W17_A | 0.2 | ND | ND | ND | ND |
| 21 | W17_B | 3.0 | ND | ND | ND | ND |
| 22 | W18 | 1.3 | ND | ND | ND | ND |
| 23 | W19 | 0.4 | ND | ND | ND | ND |
| 24 | W20 | 0.3 | ND | ND | ND | ND |
| 25 | W21 | 3.7 | ND | ND | ND | ND |
| 26 | W22 | 1.0 | ND | ND | ND | ND |
| 27 | W23 | 0.3 | ND | ND | ND | ND |
| 28 | W24 | 0.7 | ND | ND | ND | ND |
| 29 | W25_A | 4.23 | 1.2 | +++ | + | - |
| 30 | W26 | 0.8 | ND | ND | ND | ND |
| 31 | W27 | 3.1 | ND | ND | ND | ND |
| 32 | W28 | 0.2 | ND | ND | ND | ND |
| 33 | W29 | 0.1 | ND | ND | ND | ND |
| 34 | W30 | 3.9 | ND | ND | ND | ND |
| 35 | W31 | 1.4 | ND | ND | ND | ND |
| 36 | W32 | 0.1 | ND | ND | ND | ND |
| 37 | W33 | 1.7 | ND | ND | ND | ND |
| 38 | W34 | 0.2 | ND | ND | ND | ND |
| 39 | W35 | 1.9 | ND | ND | ND | ND |
| 40 | W39 | 0.2 | ND | ND | ND | ND |
| 41 | W40 | 1.3 | ND | ND | ND | ND |
| 42 | W42 | 3.6 | ND | ND | ND | ND |
| 43 | W43 | 0.3 | ND | ND | ND | ND |
| 44 | W44_A | 2.1 | ND | ND | ND | ND |
| 45 | W44_B | 2.3 | ND | ND | ND | ND |
| 46 | W45 | 0.2 | ND | ND | ND | ND |
| 47 | W46 | 0.4 | ND | ND | ND | ND |
| 48 | W47 | 1.1 | ND | ND | ND | ND |
| 49 | W48 | 0.4 | ND | ND | ND | ND |
| 50 | W49 | 1.7 | ND | ND | ND | ND |
| 51 | W50 | 0.1 | ND | ND | ND | ND |
| 52 | W52 | 2.5 | ND | ND | ND | ND |
| 53 | W52 | 0.1 | ND | ND | ND | ND |
| 54 | W63_B | 4.25 | 4.1 | ++ | + | + |

ND-not determined; For IAA production;+++ - deep pink coloration, ++ - pink coloration, +- pale red coloration; For siderophore production and ACC deaminase activity; + -positive, - negative.
