# Supplementary figures and images for: Zn-mobilizing bacteria improve shoot biomass and zinc content in wheat
Source: FEMS Microbiol Ecol. 2026 Apr 13;102(5):fiag030. doi: 10.1093/femsec/fiag030 (PMC13089531; doi:10.1093/femsec/fiag030)

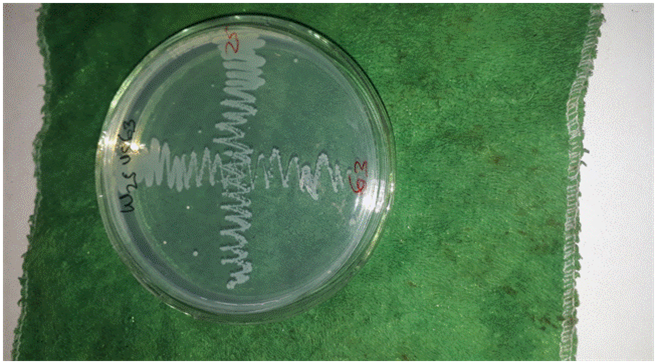


Supplementary Fig.1: a compatibility test between Zn mobilizing rhizosphere bacteria W25_A and W63_B.

Supplement: fiag030_Supplemental_Files [file fiag030_supplemental_files.zip › Supplementary Figure 1.docx]
